# bFGF plays a neuroprotective role by suppressing excessive autophagy and apoptosis after transient global cerebral ischemia in rats

**DOI:** 10.1038/s41419-017-0229-7

**Published:** 2018-02-07

**Authors:** Dawei Sun, Wenying Wang, Xintao Wang, Yan Wang, Xiaotao Xu, Feng Ping, Yu Du, Wei Jiang, Derong Cui

**Affiliations:** 1 Department of Anesthesiology, Shanghai Jiaotong University Affiliated Sixth People’s Hospital, 200233 Shanghai, China; 2 Department of Neurology, Shanghai Jiaotong University Affiliated Sixth People’s Hospital, 200233 Shanghai, China

## Abstract

Transient global cerebral ischemia (tGCI) is a cerebrovascular disorder that can cause apoptotic neuronal damage and functional deficits. Basic fibroblast growth factor (bFGF) was reported to be highly expressed in the central nervous system (CNS) and to exert neuroprotective effects against different CNS diseases. However, the effects of bFGF on tGCI have not been studied intensively. This study was conducted to investigate the effect of bFGF and its underlying mechanism in an animal model of tGCI. After intracerebroventricular (i.c.v.) injection of bFGF, functional improvement was observed, and the number of viable neurons increased in the ischemia-vulnerable hippocampal CA1 region. Apoptosis was induced after tGCI and could be attenuated by bFGF treatment via inhibition of p53 mitochondrial translocation. In addition, autophagy was activated during this process, and bFGF could inhibit activation of autophagy through the mTOR pathway. Rapamycin, an activator of autophagy, was utilized to explore the relationship among bFGF, apoptosis, and autophagy. Apoptosis deteriorated after rapamycin treatment, which indicated that excessive autophagy could contribute to the apoptosis process. In conclusion, these results demonstrate that bFGF could exert neuroprotective effects in the hippocampal CA1 region by suppressing excessive autophagy via the mTOR pathway and inhibiting apoptosis by preventing p53 mitochondrial translocation. Furthermore, our results suggest that bFGF may be a promising therapeutic agent to for treating tGCI in response to major adverse events, including cardiac arrest, shock, extracorporeal circulation, traumatic hemorrhage, and asphyxiation.

## Introduction

Transient global cerebral ischemia (tGCI) is a cerebrovascular disorder that is characterized by a transient reduction of localized blood flow to brain tissue due to either arterial obstruction or systemic hypoperfusion. This event usually occurs during adverse events such as cardiac arrest, shock, extracorporeal circulation, traumatic hemorrhage, and asphyxiation^1^. In 2015, the mortality of sudden cardiac arrest in the United States was 352,089^2^. The most effective treatment for cerebral ischemia is restoring blood flow to the affected area(s) as soon as possible. However, cerebral ischemia/reperfusion (I/R) will cause brain damage, and it has been widely reported that cerebral I/R can result in delayed neuronal death, especially in the vulnerable hippocampal CA1 region^3,4^.

Basic fibroblast growth factor (bFGF) is a well-studied member of the fibroblast growth factor family and is abundant in the central nervous system (CNS). bFGF plays important physiological and pathophysiological roles in the hippocampus during both development and adulthood. In addition, bFGF is related to neuroprotection, lesion repair, and neurogenesis and can promote neuronal plasticity in the hippocampus^5^. The protective role of bFGF in other CNS diseases, such as stroke (focal cerebral ischemia), spinal cord injury, and traumatic brain injury, has been was extensively researched, and many mechanisms have been described^6–11^. However, there is little data regarding the effect of bFGF on tGCI, and the underlying mechanism remains elusive.

There are three primary types of cell death involved in tGCI—necrosis, apoptosis, and autophagic cell death^12^; autophagy and apoptosis have been extensively studied. Autophagy is a conserved intracellular degradation pathway that traffics substrates including bulk cytoplasm, damaged organelles, long-lived proteins, and infectious agents to lysosomes^13^. Although autophagy is advantageous in most situations, grossly enhanced autophagy could contribute to “type 2” or “autophagic” cell death. Many researchers found that autophagy was activated after cerebral ischemia and that suppressing autophagy could be neuroprotective both in vivo and in vitro^14–17^. However, most models of cerebral ischemia utilize either focal cerebral ischemia in rodents or oxygen-glucose deprivation cell experiments. Our previous work demonstrated that autophagy was activated in rat hippocampus and played a bad role after cardiac arrest^18^. Thus, we wondered whether bFGF administration can elicit any changes in autophagy.

Apoptosis is characterized by distinctive morphologic changes in the nucleus and cytoplasm, chromatin cleavage at regularly spaced sites, and the endonucleolytic cleavage of genomic DNA at internucleosomal sites. p53, a well-known transcription factor, can transactivate a number of pro-apoptotic genes such as Bax, Puma, and Noxa in the nucleus and this process is characterized as “transcription-dependent p53-mediated apoptosis”. In addition to this well-established apoptosis pathway, overwhelming evidence has suggested that mitochondrial p53 translocation can provide the basis for an alternative p53-mediated apoptosis—“transcription-independent p53-mediated apoptosis”. Cytosolic p53 has no transcriptional function and can rapidly translocate to the mitochondria in response to different stresses^19^. There, it induces mitochondrial outer membrane permeabilization (MOMP) to trigger the release of signaling molecules from the intermembrane space into the cytosol and causes activation of members of the Caspase family and other processes^20^. Mitochondrial translocation of p53 was reported to mediate neuronal death after tGCI^21^, and we wondered whether bFGF could be neuroprotective through this mechanism.

In this study, we verified that our global cerebral animal model could cause transcription-independent p53-mediated apoptosis and activation of autophagy in the hippocampus. We utilized molecular biotechnology and pathological procedures to examine whether bFGF could attenuate cerebral I/R injury and the underlying mechanism involved. Furthermore, we explored the interaction between autophagy and apoptosis in this process.

## Results

### I/R triggered transcription-independent p53-mediated apoptosis and autophagy activation in the hippocampus

**Fig. 1:**
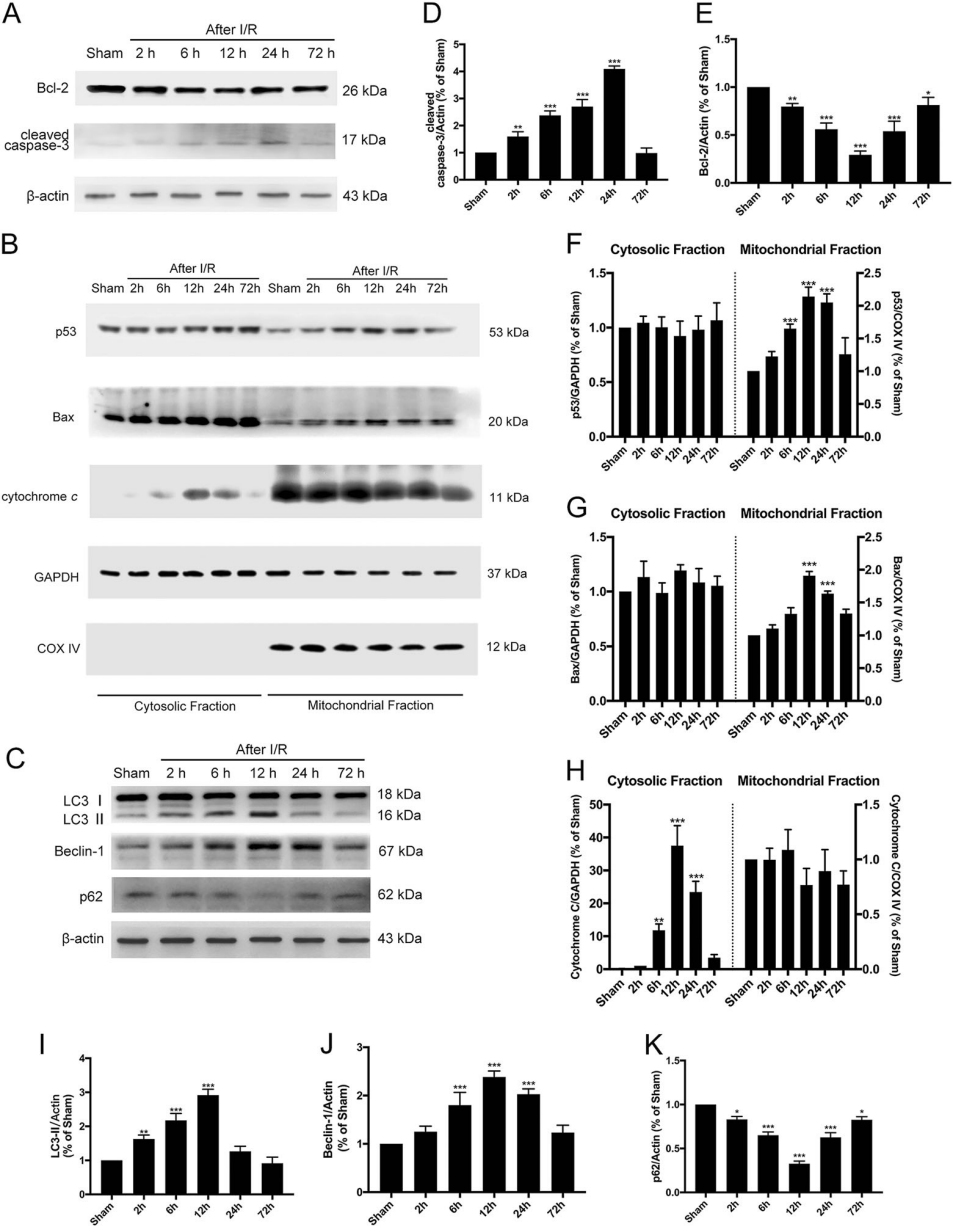
Temporal changes of autophagy and apoptosis after tGCI. **a** Immunoblots of the apoptosis-related proteins cleaved Caspase-3 and Bcl-2. Total cell lysates from the hippocampus were used, and β-actin was used as an internal control. **b** Immunoblots of p53, Bax, and cytochrome *c* in the cytosolic and mitochondrial fractions. GAPDH was used as a loading control for cytosolic proteins, and COX IV was used as the loading control for mitochondrial proteins. **c** Immunoblots of the autophagy-related proteins LC3B and Beclin-1. Total cell lysates from the hippocampus were used. **d**–**k** Densitometric analysis (mean ± SEM, *n* = 3 animals per group) of the proteins from (**a**, **b**, **c**) normalized to the respective loading controls. ^*^P < 0.05 vs. sham + vehicle, ^**^P < 0.01 vs. sham + vehicle, ^***^P < 0.001 vs. sham + vehicle

To confirm whether our animal model was successfully established, we first examined the time course of the expression of apoptosis-related proteins in the rat hippocampus. When apoptosis is induced, pro-Caspase-3 is cleaved and acts as a critical and terminal executioner^22^. In our experiment, the levels of cleaved Caspase-3 increased early after I/R and peaked at 24 h, which confirmed that I/R could cause injury (Fig. 1a). To explore whether mitochondrial p53 translocation participated in this process, we isolated the mitochondrial and cytosolic fractions of total lysate to detect temporal changes of p53, Bax, cytochrome *c*, and Bcl-2 expression in whole cell lysate (Fig. 1a–b). Mitochondrial p53 increased after I/R and began to decline 24 h after I/R; moreover, the temporal changes of Bax and Bcl-2 expression verify our hypothesis that cytosolic p53 translocates into mitochondria and collaborates with the anti-apoptotic and pro-apoptotic Bcl-2 families (i.e., Bcl-2 and Bax) to release mitochondrial cytochrome *c* into the cytosol (Fig. 1a,b, d–h). When cytosolic cytochrome *c* levels increase, the Caspase-dependent apoptosis cascade was initiated. The process of cytosolic p53 translocation into mitochondria to trigger the apoptosis signaling pathway was characterized by transcription-independent p53-mediated apoptosis.

To investigate whether autophagy was activated in the hippocampus after global cerebral I/R, we detected the time course of LC3, p62, and Beclin-1 expression (Fig. 1c). The quantitative Western blot analyses of LC3, p62, and Beclin-1 demonstrate that autophagy was activated after global cerebral ischemia and peaked at 12 h (Fig. 1i–k). The increased LC3-II expression indicated double membrane autophagosome vesicle formation, and increased Beclin-1 represented the initiation of macroautophagy. Western blotting of p62 suggested that the autophagy was complete and that autophagy flux was not impaired.

### bFGF treatment ameliorated cerebral I/R-induced injury

**Fig. 2:**
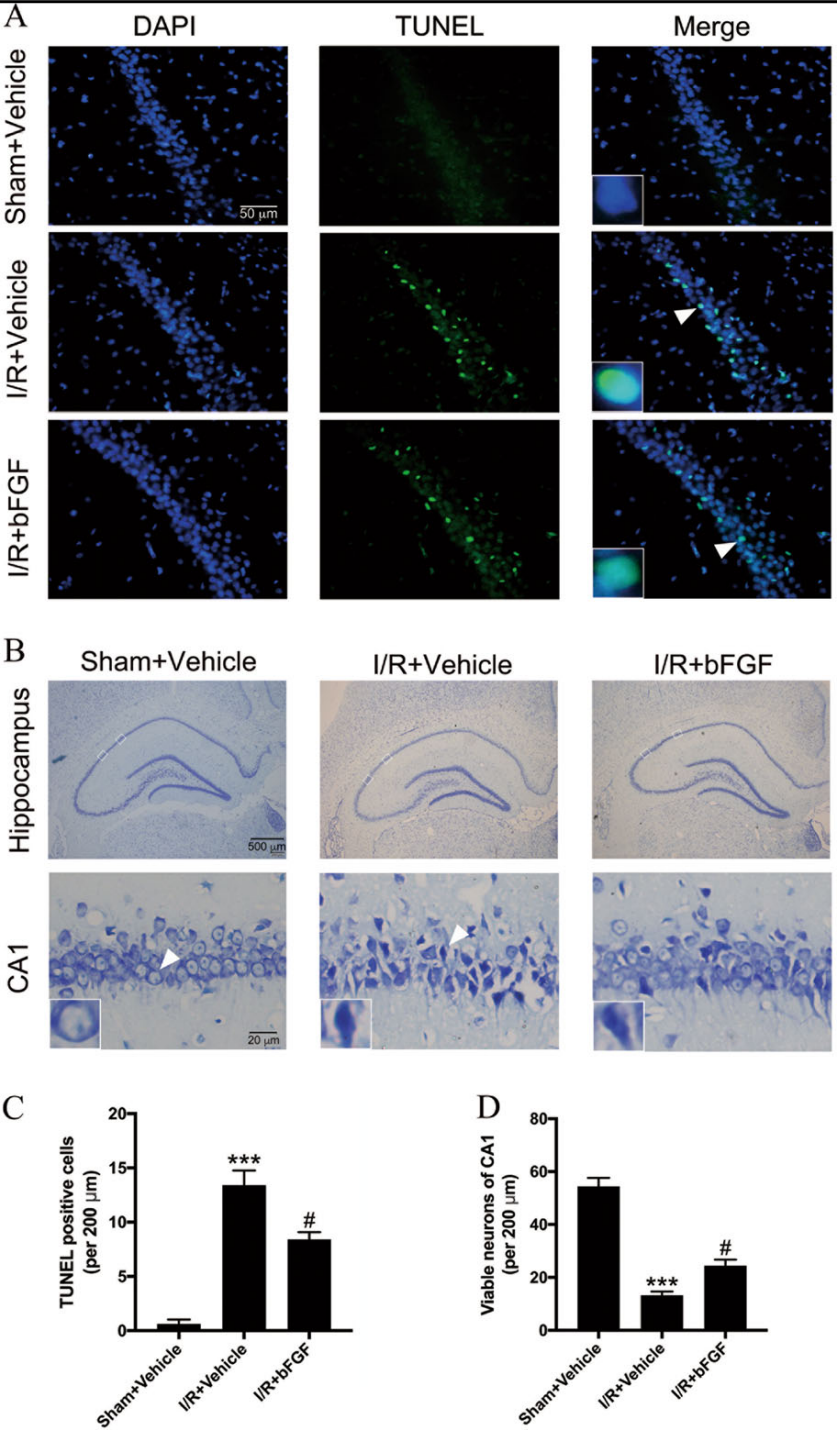
bFGF treatment generated a significant neuroprotective effect in the hippocampal CA1 region after global cerebral I/R. **a** Representative images of TUNEL staining in the CA1 region of the hippocampus 24 h after I/R. **b** Representative cresyl violet staining of the entire hippocampus and the detailed CA1 region 72 h after I/R. The white boxes in the hippocampus denote the area of the detailed CA1 region. Insets in the pictures are magnified neurons from the areas indicated by the arrowhead. **c**–**d** Quantitative data (mean ± SEM, *n* = 5–6 animals per group) of TUNEL-positive cells and viable neurons per 200-μm length of the medial CA1 region. ^***^P < 0.001 vs. sham + vehicle, ^#^*P < *0.05 vs. I/R + vehicle

**Fig. 3:**
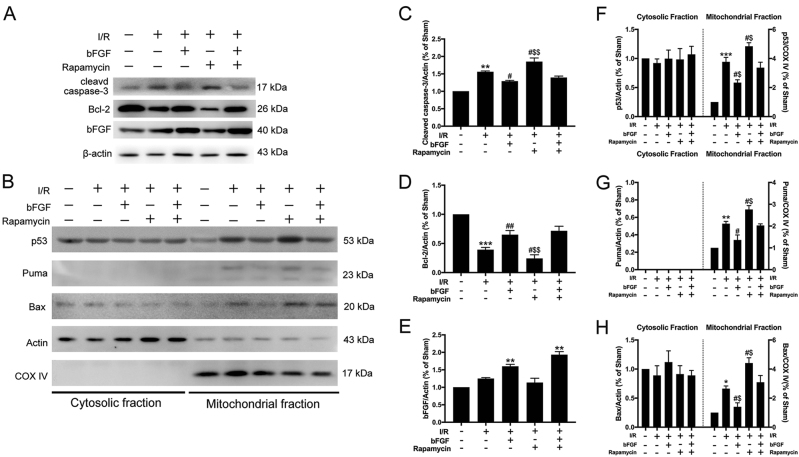
Apoptosis was decreased via inhibition of p53 translocation after bFGF treatment. **a** Immunoblots of cleaved Caspase-3, Bcl-2, and bFGF from total cell lysates of hippocampal tissue harvested 24 h after I/R. β-actin was used as an internal control. **b** Immunoblots of p53, Puma, and Bax in the cytosolic and mitochondrial fractions of samples harvested 24 h after I/R. β-actin was used as a loading control for cytosolic proteins, and COX IV was used as the loading control for mitochondrial proteins. **c**–**h** Densitometric analysis (mean ± SEM, *n* = 3 animals per group) of the proteins from (**a**, **b**) were normalized to the respective loading controls. ^**^P < 0.01 vs. sham + vehicle; ^#^*P* < 0.05, ^##^*P* < 0.01 vs. I/R + vehicle group; ^$^*P* < 0.05, ^$$^*P* < 0.01 vs. I/R + bFGF + rapamycin group

We evaluated whether bFGF treatment could be neuroprotective against global cerebral I/R in rat hippocampus. To assess apoptosis in the hippocampal CA1 region, we performed TUNEL staining 24 h after I/R. There were few TUNEL-positive cells in the sham + vehicle group but many TUNEL-positive cells in the I/R group (Fig. 2a). Quantitation of the TUNEL-positive cells per 200 μm showed that bFGF administration could attenuate I/R-induced apoptosis (Fig. 2c). Cresyl violet staining showed that at 72 h after I/R, evidence of apoptosis was visible with cell shrinkage and nuclear chromatin condensation (Fig. 2b). Quantitation of the viable neurons in the CA1 region suggests that bFGF treatment increased the number of viable neurons and played a neuroprotective role after tGCI (Fig. 2d). The immunoblotting results of cleaved Caspase-3 were also consistent with the results of the TUNEL and cresyl violet assays (Fig. 3a,c) when comparing the I/R + vehicle group with the I/R + bFGF group.

### bFGF reduced memory extinction of rats after cerebral I/R

**Fig. 4:**
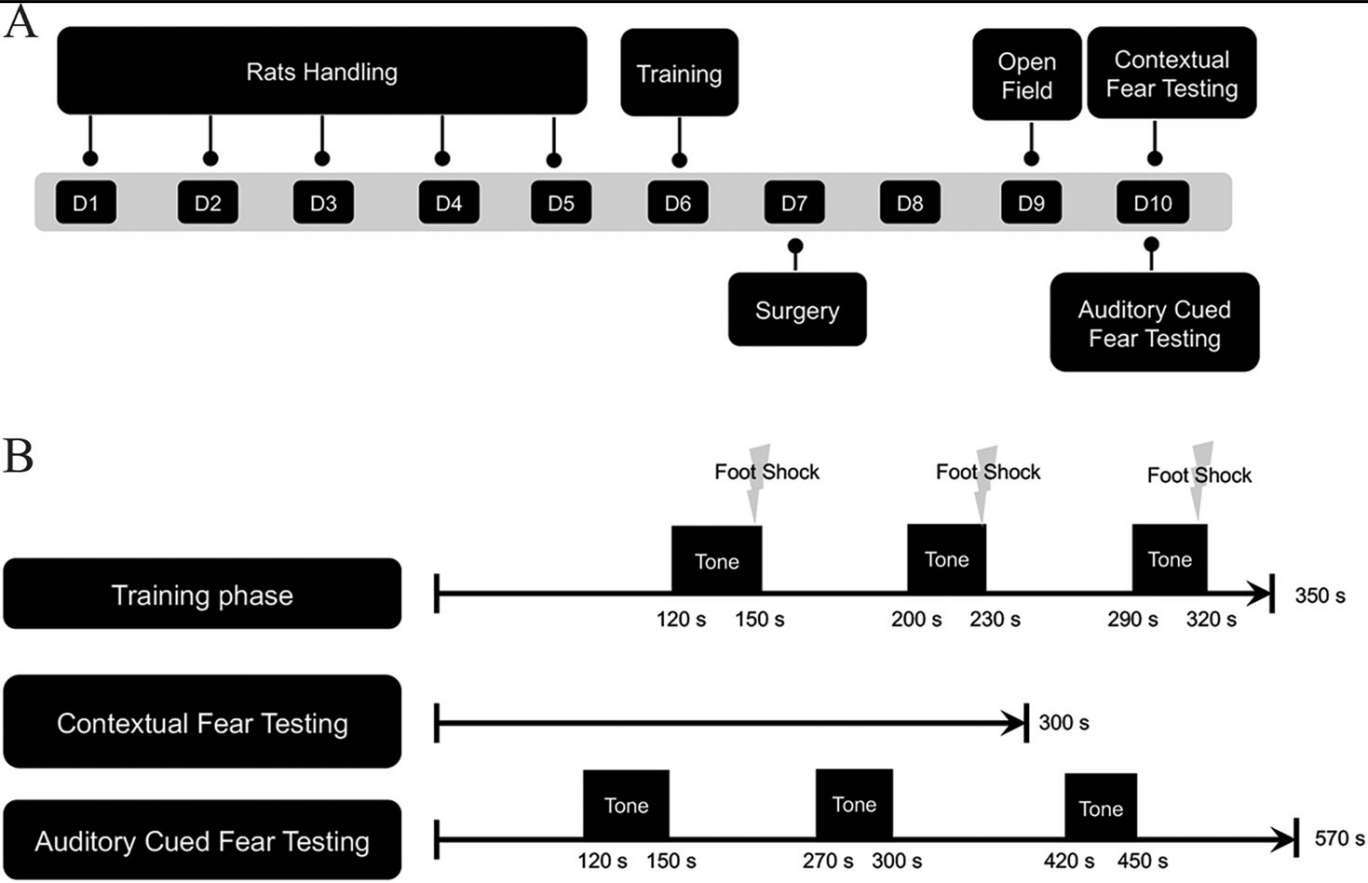
**a** Study design and timeline of the behavioral tests. **b** Schematic representation of the fear conditioning protocol

**Fig. 5:**
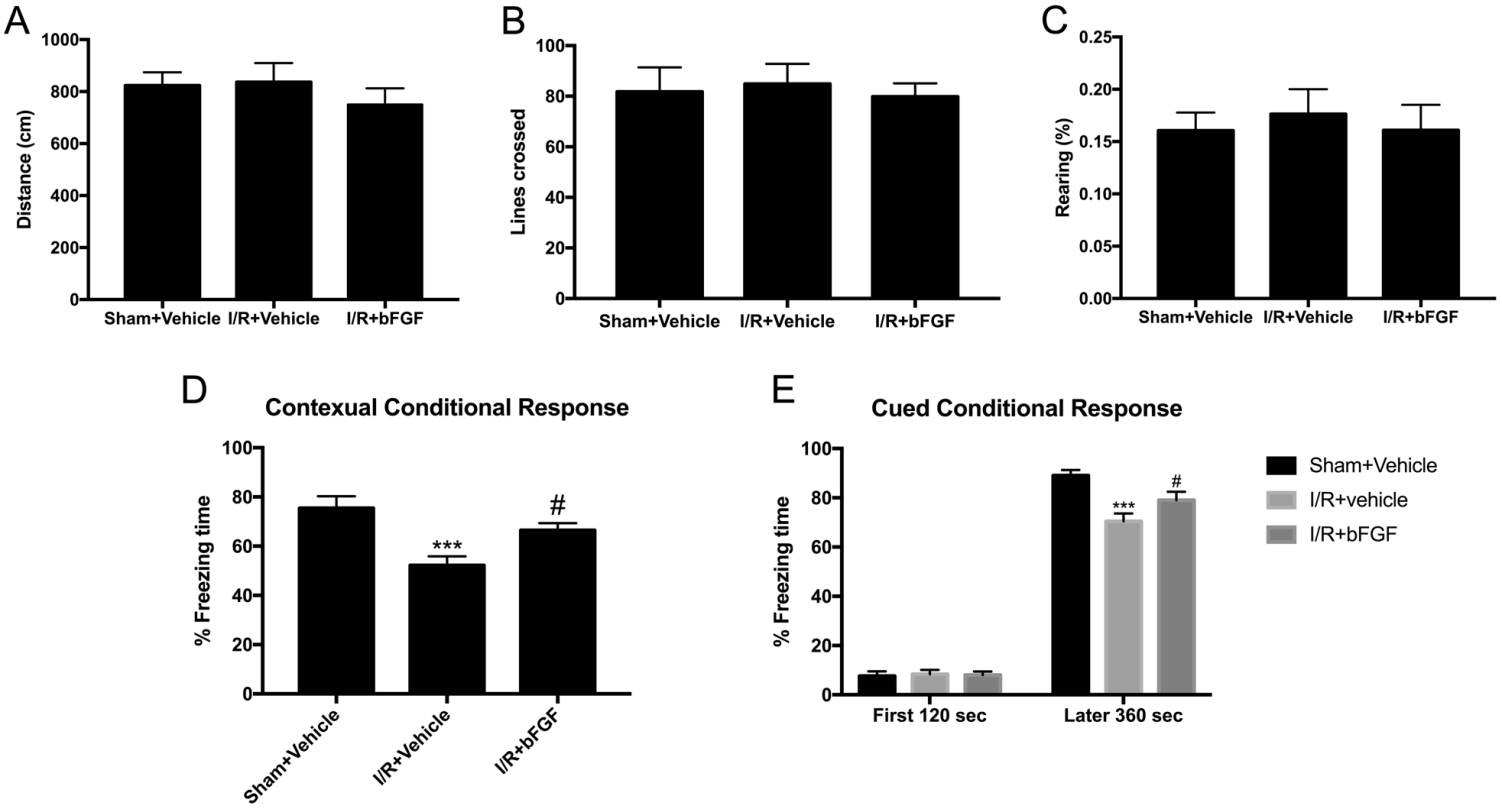
Effect of bFGF treatment on performances in the open field and fear conditioning tests. **a**–**c** Statistical results of the open field test. The open field test was implemented 2 days after surgery to assess spontaneous locomotor activity (e.g., total distance, number of lines crossed) and exploratory activity (e.g., rearing). The data are presented as the mean ± SEM. (*n* = 12 animals per group). **d** Percentage of time frozen among rats subjected to contextual fear conditioning (3 days after surgery), which reflects hippocampal-dependent memory. The data are presented as the mean ± SEM. (*n* = 12 animals per group). ^***^P < 0.001 vs. sham + vehicle. ^#^*P* < 0.05 vs. I/R + vehicle. **e** Percentage of time frozen among rats subjected to cued fear conditioning (3 days after surgery). The first 120 s was used as a baseline for comparison with the latter 360 s under auditory cued stimulus; this reflects hippocampal-independent memory. The data are presented as the mean ± SEM. (*n* = 12 animals per group). ^***^P < 0.001 vs. sham + vehicle. ^#^*P* < 0.05 vs. I/R

To investigate whether the beneficial effects of bFGF were functional, we performed fear conditioning tests to examine memory extinction (Fig. 4a). The fear conditioning paradigm we employed comprised a training phase and evaluation phase. The evaluation phase consisted of two different tests: contextual fear testing and auditory cued fear testing (Fig. 4b). Contextual fear testing examines hippocampus-dependent memory, and auditory cued fear conditioning detects hippocampus-independent memory. Before we conducted any behavioral tests (especially locomotion-related tests), we first executed an open field test to ensure the spontaneous locomotion ability of rats and to verify any differences of motion capacity after surgery in order to rule out the potential contribution of differences. The indexes we monitored were total distance, number of lines crossed and rearing. The data analyses (Fig. 5a–c) suggest that there was no difference of locomotion ability among the three groups. The contextual conditional response (Fig. 5d) showed that the percent freezing time in the I/R group significantly declined, which suggests that hippocampus-dependent memory was impaired after I/R. On the other hand, after treatment with bFGF, the rats’ behavior improved (*P* < 0.05). These results demonstrate that bFGF could not only decrease apoptosis after cerebral ischemia at the molecular level but also promote memory function. The cued conditional fear conditioning test (Fig. 5e), which was performed in a novel chamber, showed similar exploratory behavior among the three groups during the first 120 s in the non-stimulus environment. However, after encountering the familiar frightening sound, they froze dramatically. The differences among the three groups indicated that bFGF can also improve hippocampus-independent memory.

### bFGF suppressed excessive autophagy after tGCI through the mTOR pathway

**Fig. 6:**
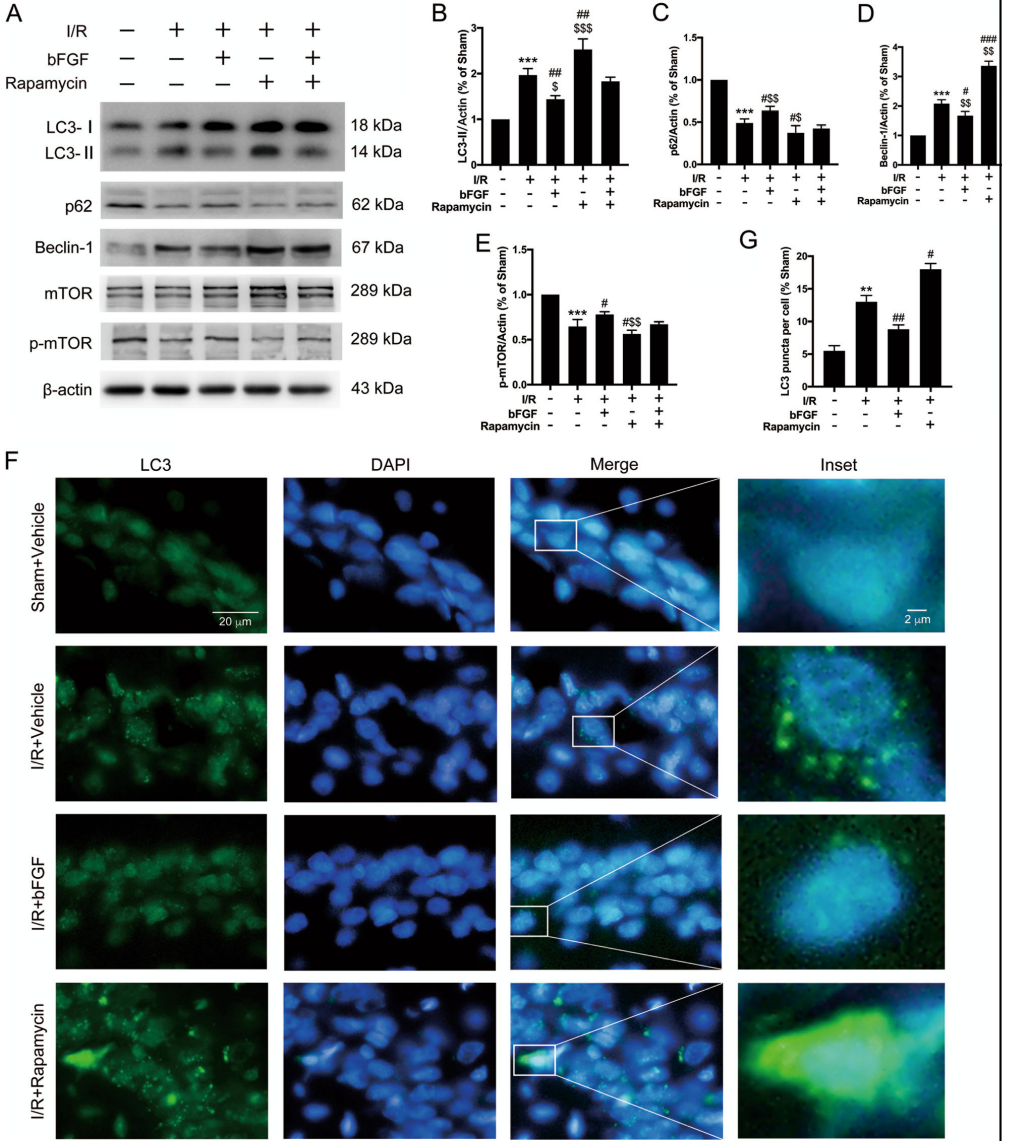
The neuroprotective effect of bFGF after global cerebral I/R functioned by restraining excessive autophagy via the mTOR pathway. **a** Immunoblots of LC3, p62, Beclin-1, mTOR, and p-mTOR was assessed with β-actin as a loading control. **b-e** Densitometric analysis (mean ± SEM, n = 3 animals per group) of the proteins from (A) normalized to the respective loading controls. **f** Representative immunofluorescence images of LC3 (green) and DAPI (blue) double staining in medial CA1 region 24 h after I/R. **g** Quantitative analysis of LC3B puncta per cell. More than 30 cells per condition were included. The data are presented as the mean ± SEM. ^***^P < 0.001 vs. sham + vehicle; ^#^*P* < 0.05, ^##^*P* < 0.01, ^###^*P* < 0.001 vs. I/R + vehicle group; ^$^*P* < 0.05, ^$$^*P* < 0.01, ^$$$^*P* < 0.001 vs. I/R + bFGF + rapamycin group

We previously discovered that autophagy was activated after global cerebral ischemia and that induced autophagy could be detrimental^18,23^; however, how bFGF affects autophagy during this process was unclear. The results of the Western blot analyses showed that the expression of LC3-II and Beclin-1 (Fig. 6a–e) were relatively declined and p62 expression was increased after bFGF administration (I/R group vs. I/R + bFGF group), which suggests that autophagy was inhibited in the hippocampus after bFGF treatment. Growth factors can activate mTORC1 to inhibit autophagy under different conditions (such as hypoxic stress, neurodegeneration, and tumorigenesis)^24,25^. To further explore whether the mTOR pathway was involved in the neuroprotection of bFGF against tGCI, we detected the protein expression levels of p-mTOR and mTOR (Fig. 6a,d). In rats administered bFGF, the levels of p-mTOR increased while mTOR showed no distinct change, which demonstrates that bFGF can upregulate p-mTOR to inhibit excessive autophagy. Immunofluorescence staining of LC3 (Fig. 6f) and its quantitative analysis (Fig. 6g) showed that the intensity of LC3 puncta increased after I/R but was decreased in the bFGF-treated group compared with the I/R group, which is consistent with immunoblotting results indicating that bFGF could inhibit autophagy. ULK1, 4E-BP1, and p70 S6 are downstream targets of mTOR and we observed decreases in their phosphorylated level after tGCI and significant increases with presence of bFGF (Supplementary Fig. 2a–f). Rapamycin is an inhibitor of mTOR complex 1, and it is widely used as an agonist of autophagy^26^. We used rapamycin as a positive control to further explore the role of mTOR during this process. When comparing the I/R group and I/R + Rapamycin group, the Western blots results for LC3, Beclin-1, and p62 (Fig. 6a) indicated that autophagy was activated after rapamycin treatment. Furthermore, the comparative results of I/R + Rapamycin with I/R+bFGF+ Rapamycin showed that the expression of LC3-II and Beclin-1 declined, whereas p62 and p-mTOR expression was increased (Fig. 6a–e). These results indicated that the rapamycin-activated autophagy could be re-inhibited by bFGF. These clues imply that the process of bFGF against excessive activation of autophagy after tGCI involves the mTOR pathway.

### bFGF protected the brain from I/R injury by inhibiting apoptosis through the p53 mitochondrial translocation pathway

**Fig. 7:**
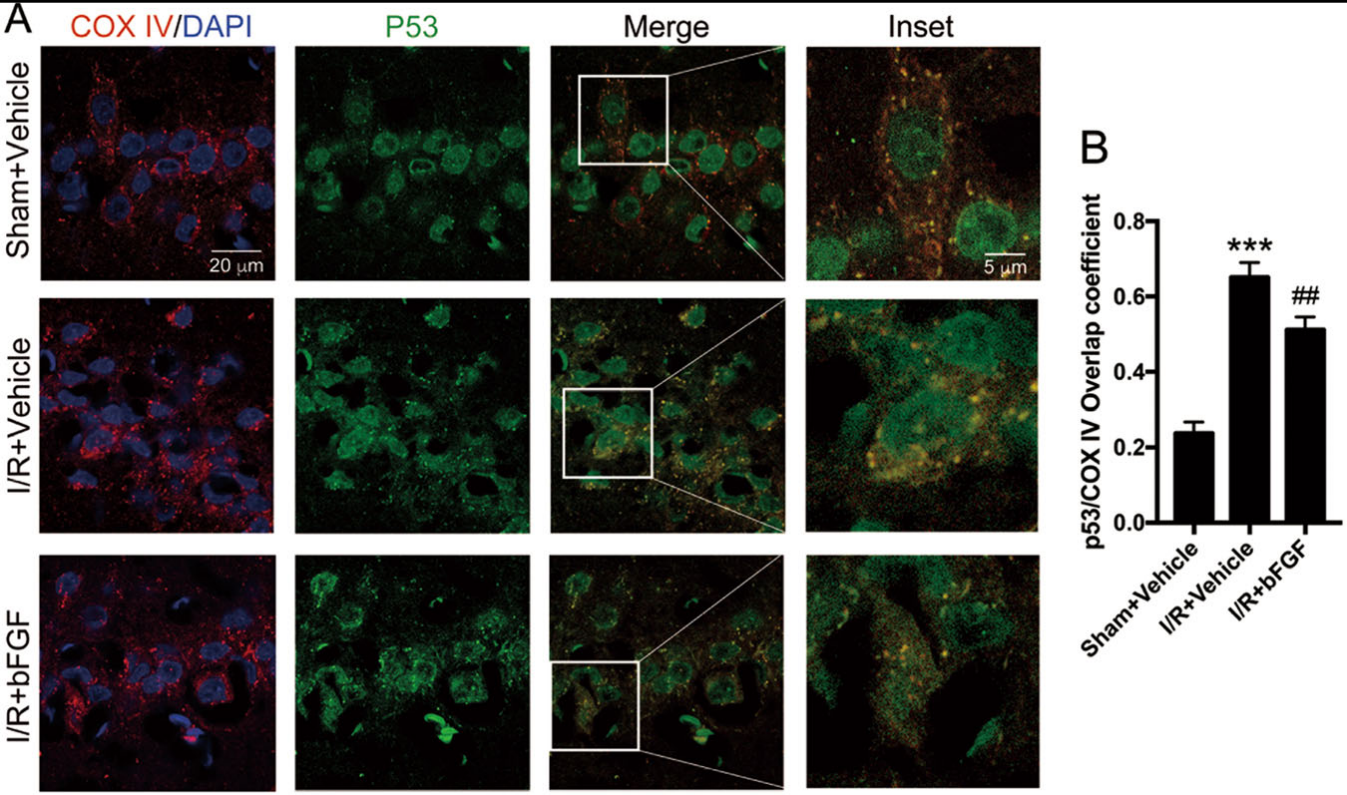
bFGF administration inhibited the translocation of p53 to mitochondria. **a** Representative confocal images of DAPI (blue), p53 (green), and COX IV (red) staining in the hippocampal CA1 region. COX IV was used as a mitochondrial marker, and nuclei were counterstained with DAPI. In the merged microphotographs, yellow puncta indicate the co-localization of p53 and mitochondria. **b** Manders’ overlap coefficient represents the quantification of co-localization of p53 and COX IV. At least ten cells from each group were selected randomly. ^***^P < 0.001 vs. sham + vehicle; ^##^*P* < 0.01 vs. I/R + vehicle group

A previous study demonstrated that mitochondrial translocation of p53 contributes to hippocampal CA1 neuronal death after global cerebral ischemia^21^, and we wondered whether the neuroprotective role of bFGF functions through this mechanism. We isolated the mitochondrial fraction from hippocampal lysates. The immunoblotting results (Fig. 3a–d) showed that the expression of the apoptotic terminal protein cleaved Caspase-3 increased notably, whereas the expression of anti-apoptotic Bcl-2 decreased after tGCI. Mitochondrial Puma and Bax expression increased after tGCI when comparing the sham + vehicle group with I/R + vehicle group (Fig. 3g–h). The treatment with bFGF was successful basing on the bFGF expression in hippocampus (Fig. 3e). Furthermore, after the treatment with bFGF, the expression of cleaved Caspase-3, mitochondrial Puma, and Bax decreased, whereas re-upregulated Bcl-2 expression was observed (Figs. 3a–d, 3g–h) when comparing the I/R group with I/R + bFGF group. These results of apoptotic markers suggest bFGF could attenuate apoptosis caused by tGCI. Interestingly, the increased mitochondrial p53 expression after tGCI was reduced by bFGF (Fig. 3b,f). We used COX IV (a mitochondrion-specific marker) for confocal immunofluorescence staining (Fig. 7a) to co-locate p53 and mitochondria, and we observed increased mitochondrial p53 immunoreactivity after I/R. The p53/COX IV overlap coefficient decreased after treatment with bFGF (Fig. 7b). We can conclude that bFGF weakened p53 mitochondrial translocation by the immunoblotting results of mitochondrial p53 and the immunofluorescence staining. These results demonstrate that the p53 mitochondrial translocation pathway was also involved in the bFGF-induced neuroprotection.

### Apoptosis caused by tGCI could be aggravated by induced autophagy and bFGF could reverse this effect

We used rapamycin treatment as a positive control to explore the interaction among bFGF, autophagy, and apoptosis in rat hippocampus after I/R. Thus, we enhanced autophagy to observe the effects of this process on I/R-induced apoptosis. The immunoblotting results for the autophagy-related proteins (Fig. 6a–f) showed that LC3-II/actin and Beclin-1 expression increased when comparing the I/R group with the I/R + Rapamycin group (the second lane vs. the forth lane). Furthermore, the expression of p62 was decreased. These results suggest that autophagy was further activated by rapamycin after tGCI. In addition, we observed increases in the immunoblots of the apoptosis-related proteins cleaved Caspase-3, and mitochondrial Bax, as well as significant decreases in Bcl-2 levels (Fig. 3a–d) by comparing I/R group vs. I/R + Rapamycin group. All these clues indicated that “enhanced autophagy can be pro-apoptosis during this process”. In contrast, when comparing the levels of autophagic and apoptotic proteins (Figs. 6a–f and 3a–d) between the I/R + rapamycin and I/R + rapamycin + bFGF groups (the right most two lanes), the changes in the band intensities suggested that “bFGF can be anti-apoptotic by suppressing excessive autophagy”. Furthermore, the Western blot results of mitochondrial Puma expression indicated that transcription-dependent p53 mediated apoptosis was also involved in this process.

## Discussion

In the CNS, bFGF is the most abundantly expressed factor among the 23 members of the fibroblast growth factor family. In the nervous system, bFGF plays a prominent role in neurogenesis, neuroprotection, differentiation during development, promotion of axonal growth, and maintenance and plasticity in adulthood^5^. The protective impact of bFGF on CNS diseases has been intensely investigated, but its impact on global cerebral ischemia is rarely studied. In the present study, we established the 2-VO global cerebral ischemia animal model to demonstrate the neuroprotective effects of bFGF after tGCI, and the underlying mechanism of this activity was related to inhibiting excessive autophagy and p53 mitochondrial translocation-mediated apoptosis.

We first examined the time course of autophagy-related and apoptosis-related proteins. Autophagy can be activated after cerebral ischemia, and in our 2-VO tGCI model, autophagy was enhanced based on the temporal detection of LC3, Beclin-1, and p62 using Western blotting and the levels of LC3 using immunofluorescence. Compared with data from reports that autophagy was impaired after traumatic brain injury^27^ and cardiac arrest (which we previously published)^18^, p62 levels were decreased after I/R and reached a minimum at 12 h, which demonstrates that autophagy flux was complete and unobstructed in the tGCI model. Cytosolic p53 protein can activate monomeric Bax by catalyzing its translocation to mitochondria and subsequent oligomerization^28^—which is known as a “hit and run” mechanism—to induce membrane permeabilization and the consequent release of cytochrome *c*. Because the translocation of p53 and Bax and the release of cytochrome *c* from mitochondria are involved in this process, we ran Western blots with mitochondrial and cytosolic fractions, respectively. Our study showed the time courses of cleaved Caspase-3, Bcl-2, p53, Bax, and cytochrome *c* levels and indicated that tGCI could induce apoptosis after I/R via p53 mitochondrial translocation. All these results provide evidence of induced autophagic cell death after tGCI.

To confirm whether i.c.v. injection could attenuate delayed neuronal death after tGCI, we next assessed brain pathology. The results showed an increased number of TUNEL-positive cells and fewer viable neurons in hippocampal CA1 region after I/R, which confirmed that I/R could cause neuronal death in the hippocampal vulnerable CA1 region. In addition, after the i.c.v. injection of bFGF, cell death could be attenuated. These data proved that bFGF exerts a neuroprotective effect against pathological changed related to I/R.

To inspect whether bFGF could lead to functional improvement after I/R, we conducted fear conditioning tests to evaluate the memory ability. The contextual conditional response reflects hippocampus-dependent memory. According to our results, hippocampus-dependent memory was impaired after cerebral I/R, which is consistent with previous reports that cerebral I/R could impair hippocampus function. The hippocampus has been well characterized regarding with memory and spatial cognition^29^. Some researchers applied the Morris water maze after cerebral I/R and found that spatial and learning memory was impaired^30,31^, whereas other laboratories used the Barnes maze^32^ and Y-maze^33^ to verify this point. The most striking difference between these mazes and fear conditioning tests is that the latter specializes on memory extinction and environmental memory rather than spatial memory. After rats were administered bFGF treatment, the memory impairments were attenuated, which indicated that bFGF could functionally protect against cerebral I/R. The auditory conditional response reflects hippocampus-independent memory, which could be related to the amygdala. In the present study, amygdala-dependent memory was impaired but could be improved with bFGF treatment based on the observations of the latter 360 s of frozen time (after receiving auditory cue); however, our primary focus concentrated on the hippocampus. Further studies are warranted to examine the effect of bFGF on the amygdala after cerebral I/R. The open field test was performed 2 days after surgery to detect the spontaneous locomotive ability of the rats and aimed to verify any differences in motion capabilities after surgery and exclude the potential contribution of the surgical procedure on the differences in activity. Our results showed no significant differences among the three groups based on total distance, number of lines crossed and rearing. In contrast, some reports showed hyperactivity after cerebral I/R^32^, but some reports are in line with our results^34,35^. These distinctions may be attributed to differences in the severity of brain damage in the animal model used or the different time points of the open field test after surgery^35^.

bFGF could ameliorate injury after tGCI from at both the molecular and functional levels, and we further explored its underlying mechanism. Autophagy was enhanced after tGCI, but whether this activity was detrimental or beneficial was unknown. Autophagy is a double-edged sword in numerous diseases, as well as in cerebral ischemia^36^. For example, Wen et al. demonstrated that 3-MA, an inhibitor of autophagy, could reduce infarct volume after permanent occlusion of the middle cerebral artery (pMCAO)^37^. Zhao, Y. N. et al. showed that intermittent hypoxia preconditioning could exacerbate neuronal injury by activating autophagy via the mTOR pathway in a 4-VO tGCI model^38^. In contrast, Michalis Papadakis et al. revealed that autophagy could be protective against ischemia of the CA3 region using a global cerebral ischemia model^39^. In addition, Wang P et al. demonstrated that nicotinamide phosphoribosyltransferase promotes neuronal survival by inducing autophagy during in vivo and in vitro models of cerebral ischemia^40^. In addition, we have previously reported that autophagy plays an antagonistic role in global cerebral I/R^18,23,41^. In the present study, autophagosome accumulation occurred after cerebral I/R, apoptosis was aggravated and p-mTOR expression decreased. Therefore, rapamycin, an mTOR inhibitor, was used as an activator of autophagy to explore the effects of excessive autophagy activation on neuronal apoptosis after global cerebral I/R. The expression levels of cleaved Caspase-3, Bcl-2, and Bax revealed that apoptosis was further aggravated in the rapamycin treatment group compared with the I/R-only group, which indicated that excessive autophagy contributed to apoptosis in global cerebral I/R. In addition, when comparing the I/R + bFGF and I/R-only groups or the I/R + rapamycin and I/R + rapamycin + bFGF groups, we concluded that “bFGF could inhibit excessive autophagy via the mTOR pathway“ as assessed by measuring the expression of biomarkers associated with autophagy and apoptosis. But we did not observe significant increases in all of the apoptotic markers when comparing I/R + bFGF with I/R + bFGF + rapamycin. We speculate the cause is certain dose of rapamycin might not contribute to significant changes of apoptotic markers with the presence of bFGF.

Hidenori Endo et al. reported that mitochondrial translocation of p53 mediates hippocampal CA1 neuronal death^21^; based on this observation, we hypothesized that transcription-independent p53-mediated apoptosis may be involved in the neuroprotective effects because of the similarity of our animal models. Thus, we isolated the mitochondrial fraction from total lysate to examine changes in mitochondrial p53 and performed immunofluorescence staining to observe colocalization of p53 with mitochondria. These results demonstrated that “the levels of mitochondrial p53 increased after cerebral I/R and that bFGF could reverse this tendency”. The expression levels of cleaved Caspase-3, Bcl-2, and mitochondrial Bax further progress the activity this pathway. Puma, which is the abbreviation for p53 upregulated modulator of apoptosis, can activate Bax and Bak to trigger the release of apoptotic factors. Puma expression increased after cerebral I/R, which indicated that transcription-dependent p53-mediated apoptosis occurred during this process—an observation consistent with that in a previous study^42^.

The immunoblotting results for bFGF suggest that endogenous bFGF was increased after cerebral I/R and that our i.c.v. injection of bFGF was successful. However, i.c.v. injection is an invasive procedure, and peripheral administration of bFGF could not reproduce the observed moderate neuroprotection due to the low efficiency bFGF transport across the blood—brain barrier^43^. Furthermore, clinical trials have demonstrated that high dosed of bFGF could cause adverse effects^44^. Therefore, many researchers sought to develop new methods to improve the delivery of bFGF to the brain, for example, intranasal administration of bFGF using nanoliposomes^11,45^, heparinized chitosan/poly(g-glutamic acid) nanoparticle delivery of bFGF^46^, and heparin-mimicking polymer conjugate to stabilize bFGF^47^. In addition, further study is needed to explore the effect of biomaterials combined with bFGF on the treatment of tGCI. Another limitation of our experiment is the lack of extended periods of bFGF administration and short observation periods after tGCI due to the tremendous effort required. Furthermore, mTOR pathway regulates many other pathways in addition to autophagy. Autophagy’s pro-death effect was one of the possibilities and there may be other mechanism involved in this process. This need to be elaborated in the future.

In conclusion, the present study demonstrates that global cerebral I/R could activate autophagy via the mTOR pathway and induce apoptosis via mitochondrial p53 translocation in the hippocampal CA1 region. Excessive autophagy contributed to apoptosis after cerebral I/R, and i.c.v. injection of bFGF could suppress excessive autophagy and apoptosis to exert neuroprotective effects and lead to functional improvement. Taken together, our results indicate that bFGF may be a promising therapeutic agent against tGCI-induced brain injury.

## Materials and methods

### Global cerebral ischemia

Fifteen minutes of global cerebral ischemia was induced by occlusion of the bilateral common carotid arteries concomitant with hypotension as previously described with some modifications^23,41,48,49^. Eight-month-old Male Sprague-Dawley rats weighing 250–300 g were used. Rats were put to fast for 8–12 h before surgery to normalize their blood glucose. After the rats were intraperitoneally (i.p.) administered 40 mg/kg 1.5% (w/v) pentobarbital sodium, they were fixed on an electronic homeothermic board set to 37 °C; the rectal temperature was measured using a probe controlled by the board. The tail artery was cannulated to monitor the mean arterial pressure (MAP). After a middle neck skin incision was made, the right jugular vein and bilateral common carotid arteries were isolated. The right jugular vein was then cannulated for drug administration and blood withdrawal to control the MAP between 40–50 mmHg, which was performed by either withdrawing or injecting blood to induce hypotension. When the MAP reached hypotensive levels, the bilateral common carotid arteries were clamped with aneurysm clips. After 15 min of occlusion, blood was reinjected, and the common carotid arteries were unclamped.

All the rats were supplied by the Experimental Animals Center of Shanghai Jiaotong University (Shanghai, China). All procedures complied with the National Institute of Health Guide for the Care and Use of Laboratory Animals and were approved by the Animal Research Committee of Shanghai Jiaotong University. Furthermore, all experiments were reported in compliance with the ARRIVE guidelines. All efforts were made to reduce the animal’s pain and minimize the total number of animals used in the study.

### Drug administration

bFGF (#100-18B, Peprotech, Rocky Hill, CT, USA) was dissolved in phosphate-buffered saline (PBS) (containing 5% alginate) at a final concentration of 0.1 mg/ml. Rapamycin (V900930, Sigma Aldrich, St. Louis, MO, USA) was dissolved in 0.1% DMSO at a final concentration of 7 μmol/L. Either bFGF (0.5 μg in 5 μl) or rapamycin (35 pmol in 5 μl) was infused into the left cerebral ventricle 30 min after tGCI onset. All rats in the same experiment received an equal volume of the vehicle. A cranial burr hole was made close to the left coronal suture, and the drugs were delivered using a 26-gauge needle protruding below the dura into the third lateral ventricle of the left hemisphere (coordinates: posterior −1.1 mm, medial/lateral −1.5 mm relative to bregma, dorsal/ventral −4 mm below dura). The injection rate was 1 µl/min, and the injection needle was left in place for 2 min after injection to allow complete dispersion of the solution.

### Behavioral testing

Fear conditioning test (FreezeFrame 4, ActiMetrics, Wilmette, IL) was used to conduct extinction trials to evaluate hippocampus-dependent and hippocampus-independent memory^50–52^. Fear conditioning consists of a training phase prior to surgery and an evaluation phase after surgery.

One day before surgery, some of the rats were subjected to a contextual fear conditioning training phase. Rats were allowed to explore the chamber for 120 s to acclimate to the environment. Then, they were subjected to 30 s of a 75-dB auditory cue (conditioned stimulus, CS) that co-terminated with a 0.6-mA 2 s foot shock (unconditioned stimulus, US). The intervals between the US and CS were random and ranged from 45 s to 60 s but were standardized for all the rats. After 3 pairs of CS-US, the rats were left in the training chamber for 30 s and then removed into new cages to prevent disturbing the previous cage mates. The chambers were wiped thoroughly with 75% ethanol spray between each animal. The rats were monitored throughout the training phase for defensive freezing posture, which is defined as the cessation of all movement except breathing.

The evaluation phase comprised contextual fear conditioning for hippocampus-dependent memory and cued fear conditioning for hippocampus-independent memory. These tests were conducted 3 days after surgery. For contextual fear testing, a rat was placed in the chamber for 5 min with no tone or shock presentation. Then, the chamber was cleaned thoroughly. For cued condition testing, the rats were placed into a novel chamber, which had different smell and different brightness 2 h after undergoing the contextual fear test. Three auditory tones separated by 120-s intervals were played. The freezing time during the contextual 5-min period and the tune-cued 120-s intervals was recorded. The freezing threshold was determined by an individual animal’s motion index histogram. Memory function and extinction trial were measured as the percentage of time frozen during the total time observed.

Two days after surgery, rats were assessed for spontaneous locomotion in the open field test. Rats were observed for 5 min, and the total distance, the number of lines crossed and rearing were used as indicators of motor activity. All experiments were performed during the light cycle between 8:00 a.m. and 11:30 a.m. All the rats were handled for 2–3 min per day for 5 consecutive days before any behavioral procedure to eliminate handling stress as a confounding variable. All the animals were placed in the behavioral testing room 30 min before training and testing to allow for habituation to the environment.

### Protein preparation and immunoblotting

At different time points, the rats were deeply anesthetized with 500 mg/kg chloral hydrate and transcardially perfused with 4 °C saline. The hippocampus was harvested quickly and homogenized as described previously^23^. Briefly, to extract total protein, tissue samples were homogenized in cold protein extraction reagent (#78503, Thermo Fisher, Rockford, IL) containing 1% protease inhibitor (#78429, Thermo Fisher, Rockford, IL) and 1% phosphatase inhibitor (#P0044, Sigma Aldrich) if necessary. Then, the samples were centrifuged at 14,000×*g* for 10 min at 4 °C, and the supernatants were collected. For cytosolic and mitochondrial protein extraction, an isolation kit (C3606, Beyotime Technology, China) was used according to the manufacturer’s instructions. The protein concentrations of these samples were analyzed using a BCA kit (P0009, Beyotime Technology), and equal amounts of protein (15–50 μg) were loaded onto 10 or 12% SDS-polyacrylamide gels. After the proteins were separated, they were transferred from the gel to a PVDF membrane, which was blocked with 5% nonfat dry milk and 1% BSA (V900933, Sigma Aldrich) in TBST (0.1% Tween in Tris-buffered saline) for 1 h at room temperature. The membranes were then incubated with appropriate primary antibodies (listed below) at 4 °C, after which they were washed and incubated with an appropriate dilution (1:1000–1:5000) of either goat anti-rabbit or goat anti-mouse secondary antibodies for 2 h at room temperature. Protein bands were detected using ImageQuant LAS 4000 (General Electric Company, USA) with an enhanced ECL substrate (#34080, Thermo Fisher). The results were analyzed and quantified using ImageJ software (version 2.0.0, USA), and the densitometric values were normalized based on the GAPDH/ β-actin/COX IV immunoreactivity to normalize the loading amounts.

The following antibodies were used for immunoblotting: rabbit anti-GAPDH (1:1000, #5174), rabbit anti-p-mTOR (1:1000, #2974), rabbit anti-mTOR (1:1000, #2983), rabbit anti-cleaved Caspase-3 (1:250, #9664), mouse anti-p53 (1:1000, #2524), rabbit anti-Puma (1:200, #7467), rabbit anti-p-4E-BP1 (1:1000, #2855), rabbit anti-4E-BP1 (#9644), rabbit anti-p-p70 S6 (1:1000, #9234), rabbit anti-p70 S6 (1:1000, #2708), rabbit anti-p-ULK1 (1:1000, 14202), rabbit anti-ULK1 (1:1000, #8054), and rabbit anti-COX IV (1:1000, #4850) from Cell Signaling Technology (Danvers, MA, USA); rabbit anti-LC3 (1:2000, ab51520), rabbit anti-p62 (1:1000, ab109012), rabbit anti-cytochrome *c* (1:2000, ab133504), and rabbit anti-Bax (1:800, ab32503) from Abcam (Cambridge, MA, USA); mouse anti-bFGF (1:1000, sc365106), rabbit anti-Beclin 1 (1:1000, sc-11427), and rabbit anti-Bcl-2 (1:1500, sc-783) from Santa Cruz Biotechnology, Inc. (Santa Cruz CA, USA); and mouse anti-β-actin (1:1000, EM21002), goat anti-rabbit secondary antibody (1:5000, HA1001-100), and goat anti-mouse secondary antibody (1:4000, HA1006) from Hangzhou Huaan Biotechnology (Hangzhou, China).

### Nissl’s staining and neuron counts

After the rats were anesthetized with 500 mg/kg chloral hydrate, they were transcardially perfused with ice-cold (4 °C) 1× PBS followed by 4% paraformaldehyde (PFA) in 1× PBS. Brains were carefully removed and immersed in PFA for 2 h. Then, the brains were processed for paraffin embedding and were sliced into 5-μm sections. For histologic assessment, every third section within a group of fifteen was stained with 0.01% cresyl violet (C0117, Beyotime Technology) and mounted. The number of neurons was counted bilaterally (five slides per animal). Cell counts from the right and left hippocampus on each of the five slides were averaged to provide a mean value.

### Immunohistochemistry and TUNEL staining

Anesthetized rats were processed at the specified time points and successively underwent transcardiac perfusion of ice-cold PBS and 4% PFA in PBS. Brains were quickly removed, post-fixed in 4% PFA overnight at 4 °C and dehydrated in a 20/30% sucrose gradient until the brains sank. Frozen sections from these brains (10-μm thickness) were sliced and mounted on slides. The slides were blocked with 5% BSA (V900933, Sigma Aldrich) in TBS containing 0.1% Triton X-100 (X100, Sigma Aldrich) for 1 h at room temperature. After the slides were incubated overnight with primary antibodies, they were incubated with secondary antibodies for 2 h at room temperature. the nuclei were stained with DAPI (#4083, Cell Signaling Technology) for 3 min at temperature, and the slides were stored at 4 °C away from light.

The primary antibodies used for immunohistochemistry were rabbit anti-LC3 (1:250, ab51520, Abcam), rabbit anti-COX IV (1:500, ab16056, Abcam), and mouse anti-p53 (1:2000, #2524, Cell Signaling Technology). The secondary antibodies goat anti-mouse IgG H&L (Alexa Fluor^®^ 488) (1:2000, ab150113) and goat anti-rabbit IgG H&L (Alexa Fluor^®^ 594) (1:2000, ab150080) were purchased from Abcam (Cambridge, MA, USA).

TUNEL staining was conducted on frozen brain slides using a TUNEL Apoptosis Assay Kit (#C1086, Beyotime Technology) in accordance with the manufacturer’s manual. The count of the TUNEL-positive cells matched the Nissl’s staining neuron count.

Microscopy images were acquired using a Leica DM IL LED inverted microscope (Buffalo Grove, IL, USA) and Zeiss LSM 710 Confocal Microscope (Athens, GA, USA). The images were captured at ×40 magnification with an inverted fluorescence microscope or ×100 with confocal microscope. The applied emission wavelengths applied were 620 nm (Alexa Fluor-594), 535 nm (Alexa Fluor-488), and 460 nm (DAPI). The exposure times were controlled for all sections in each experiment. Images were further quantified using ImageJ 2.0.0 software (National Institutes of Health, Bethesda, MD) and Image Pro Plus 6.0 (Media Cybernetics Inc, Rockville, MD). Five randomly selected fields from the bilateral hippocampal CA1 region of one coverslip were included and the experiments were repeated independently at least 3 times. Total numbers of LC3B puncta per cell were quantified using ImageJ and the Analyze Particles Plugin plus Watershed algorithm filter (a constant threshold for all of the images within each experiment was applied). Thirty cells per condition were included for quantification.

### Statistical analysis

Statistical analyses were performed using SPSS (version 23, IBM SPSS Inc., Chicago, IL, USA) and GraphPad Prism (version 7.0a, GraphPad Software Inc., La Jolla, CA, USA) software. One-way ANOVA followed by Dunnett’s post hoc test was used to compare the sham + vehicle group with the other experimental groups at various time points. One-way ANOVA followed by Tukey’s post hoc test was employed to determine significant differences between groups. The results of the cued conditional response were analyzed using two-way ANOVA followed by Tukey’s post hoc test. All *P*-values <0.05 were considered statistically significant, and all statistical results are presented as the mean ± SEM.

## Electronic supplementary material

Supplementary Figure 1

Supplementary Figure 2

Supplementary Information
