# Supplementary figures and images for: bFGF plays a neuroprotective role by suppressing excessive autophagy and apoptosis after transient global cerebral ischemia in rats
Source: Cell Death Dis. 2018 Feb 7;9(2):172. doi: 10.1038/s41419-017-0229-7 (PMC5833346; doi:10.1038/s41419-017-0229-7)

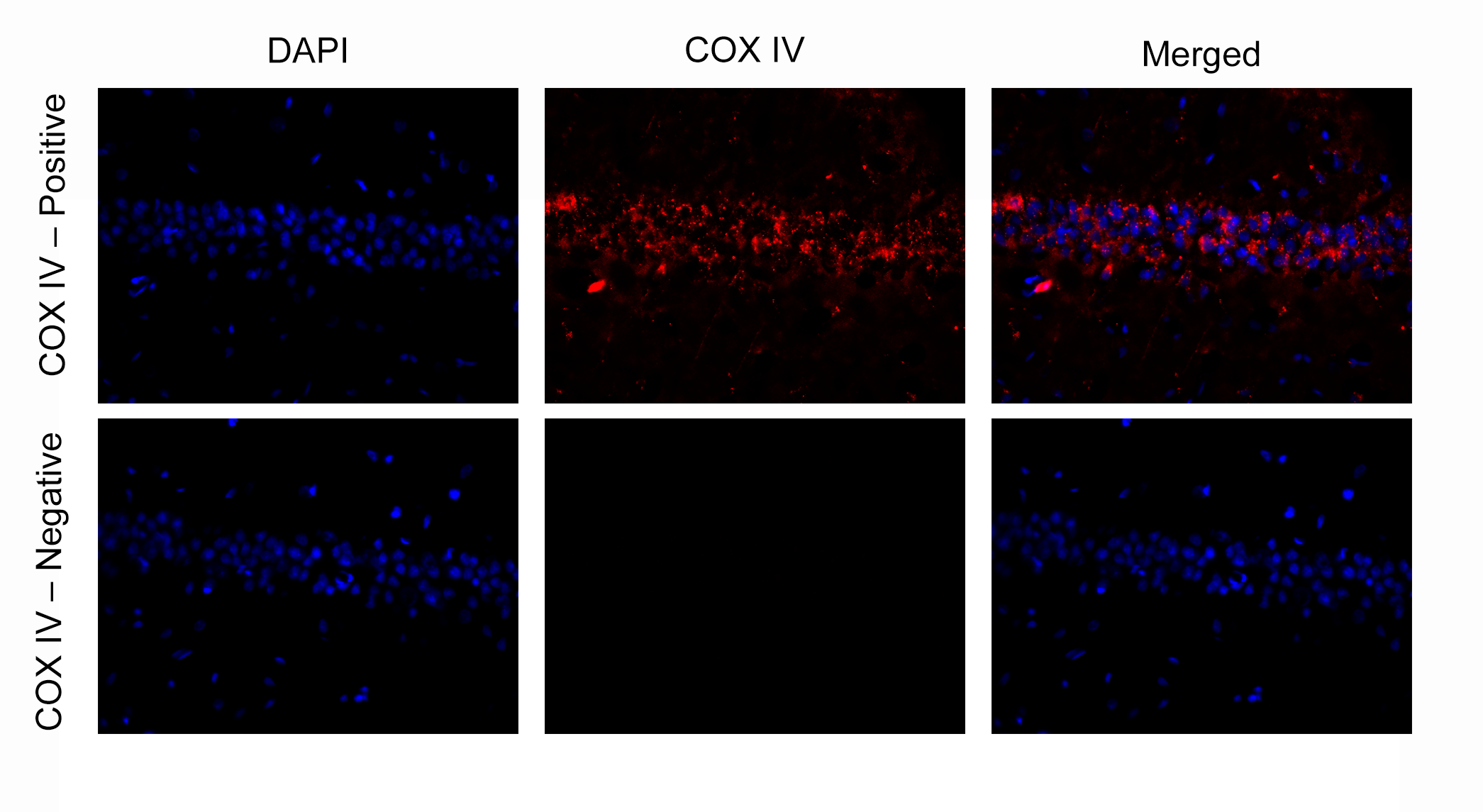

Supplement: Supplementary file 1 — Supplementary Figure 1 [file 41419_2017_229_MOESM1_ESM.tif]

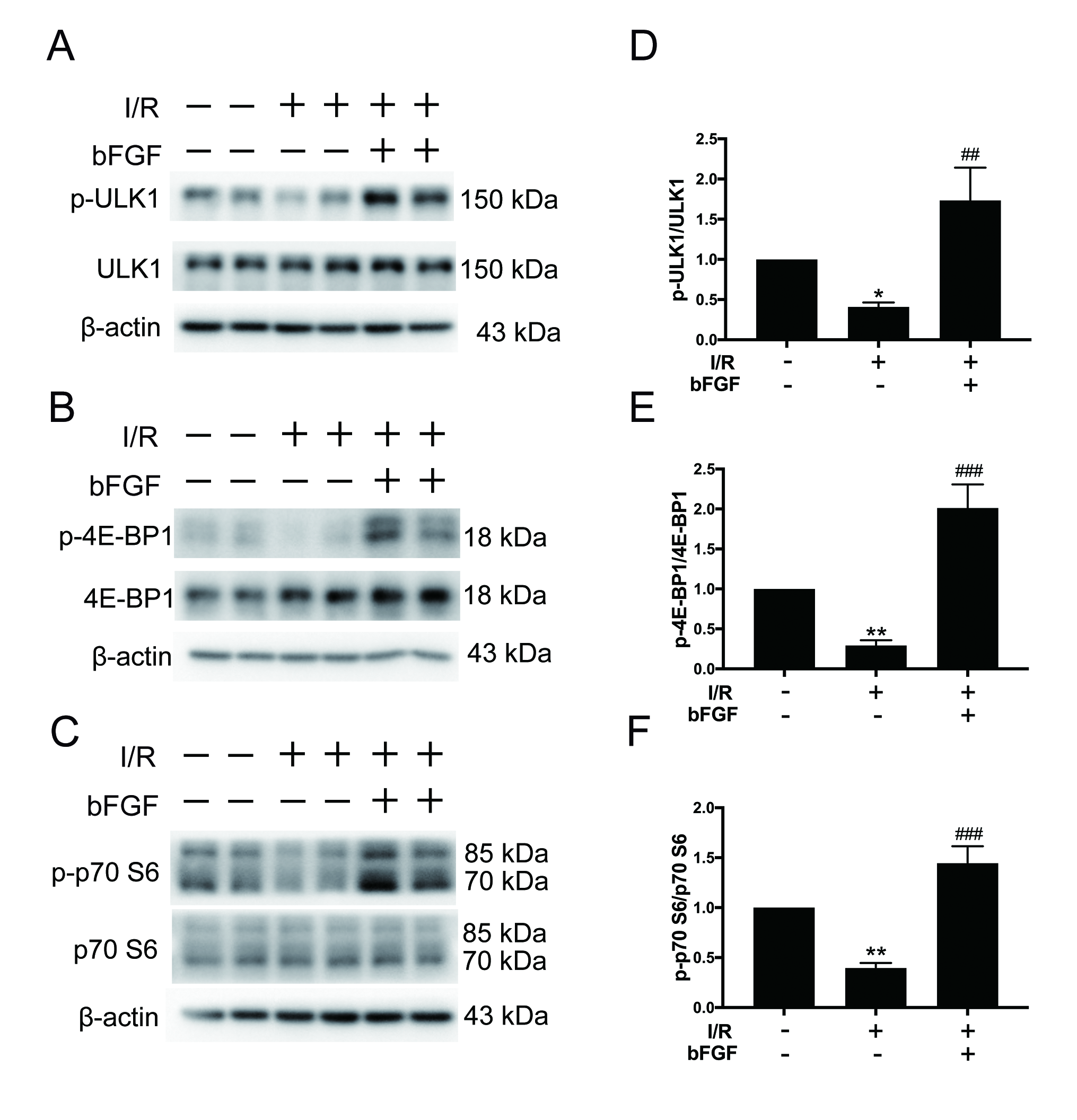

Supplement: Supplementary file 2 — Supplementary Figure 2 [file 41419_2017_229_MOESM2_ESM.tif]
