# Supplementary material for: bFGF plays a neuroprotective role by suppressing excessive autophagy and apoptosis after transient global cerebral ischemia in rats
Source: Cell Death Dis. 2018 Feb 7;9(2):172. doi: 10.1038/s41419-017-0229-7 (PMC5833346; doi:10.1038/s41419-017-0229-7)
Supplement: Supplementary file 3 — Supplementary Information [file 41419_2017_229_MOESM3_ESM.docx]

**Supplemental Figure Legends**

**Supplementary Fig.1** Negative control for COX IV immunofluorescence. In order to confirm the specific staining of COX IV, we performed the negative control for COX IV.

**Supplementary Fig.2** The change of mTOR downstream targets after tGCI with or without bFGF. **(a-c)** Immunoblots of mTOR downstream targets ULK1, 4E-BP1 and p70 S6 were assessed and actin was used as loading control. Densitometric analysis (mean±SEM, n=4 animals per group) of ratio of phosphorylated versus total protein level of proteins from (A-C). ^*^*P*<0.05 versus sham+vehicle, ^**^*P*<0.01 versus sham+vehicle, ^##^*P*<0.01 versus I/R+vehicle group, ^###^*P*<0.001 versus I/R+vehicle group
